# Focused Ultrasound-Mediated Disruption of the Blood–Brain Barrier for AAV9 Delivery in a Mouse Model of Huntington’s Disease

**DOI:** 10.3390/pharmaceutics16060710

**Published:** 2024-05-24

**Authors:** Bernie S. Owusu-Yaw, Yongzhi Zhang, Lilyan Garrett, Alvin Yao, Kai Shing, Ana Rita Batista, Miguel Sena-Esteves, Jaymin Upadhyay, Kimberly Kegel-Gleason, Nick Todd

**Affiliations:** 1 Department of Radiology, Brigham and Women’s Hospital, Harvard Medical School, Boston, MA 02115, USA; yongzhiz@bwh.harvard.edu (Y.Z.); ntodd1@bwh.harvard.edu (N.T.); 2 College of Science, Northeastern University, Boston, MA 02115, USA; garrett.l@northeastern.edu; 3 Department of Engineering, Harvard University, Cambridge, MA 02138, USA; ayao@college.harvard.edu; 4 Department of Neurology, Massachusetts General Hospital, Harvard Medical School, Charlestown, MA 02129, USA; kshing@mgh.harvard.edu (K.S.); kkegelgleason@mgh.harvard.edu (K.K.-G.); 5 Department of Neurology, University of Massachusetts Chan Medical School, Worcester, MA 01655, USA; rita.batista@umassmed.edu (A.R.B.); miguel.esteves@umassmed.edu (M.S.-E.); 6 Department of Anesthesiology, Critical Care and Pain Medicine, Boston Children’s Hospital, Harvard Medical School, Boston, MA 02115, USA; jaymin.upadhyay@childrens.harvard.edu

**Keywords:** focused ultrasound, microbubbles, blood–brain barrier opening, gene therapy, Huntington’s disease

## Abstract

Huntington’s disease (HD) is a monogenic neurodegenerative disorder caused by a cytosine–adenine–guanine (CAG) trinucleotide repeat expansion in the *HTT* gene. There are no cures for HD, but the genetic basis of this disorder makes gene therapy a viable approach. Adeno-associated virus (AAV)-miRNA-based therapies have been demonstrated to be effective in lowering HTT mRNA; however, the blood–brain barrier (BBB) poses a significant challenge for gene delivery to the brain. Delivery strategies include direct injections into the central nervous system, which are invasive and can result in poor diffusion of viral particles through the brain parenchyma. Focused ultrasound (FUS) is an alternative approach that can be used to non-invasively deliver AAVs by temporarily disrupting the BBB. Here, we investigate FUS-mediated delivery of a single-stranded AAV9 bearing a cDNA for GFP in 2-month-old wild-type mice and the zQ175 HD mouse model at 2-, 6-, and 12-months. FUS treatment improved AAV9 delivery for all mouse groups. The delivery efficacy was similar for all WT and HD groups, with the exception of the zQ175 12-month cohort, where we observed decreased GFP expression. Astrocytosis did not increase after FUS treatment, even within the zQ175 12-month group exhibiting higher baseline levels of GFAP expression. These findings demonstrate that FUS can be used to non-invasively deliver an AAV9-based gene therapy to targeted brain regions in a mouse model of Huntington’s disease.

## 1. Introduction

Huntington’s disease (HD) is a trinucleotide repeat expansion disorder. This hereditary neurodegenerative disease is caused by cytosine–adenine–guanine (CAG) trinucleotide repeats in the first exon of the *HTT* gene that encodes Huntingtin (HTT). This mutation results in the production of mutant HTT (mHTT), which has an expanded polyglutamine (polyQ) domain near the N-terminus of the protein, due to an increase in the number of polyQ-encoding CAG repeats [1,2,3,4]. MHTT forms aggregates in the cytoplasm and nucleus of neurons and is associated with a cascade of pathogenic processes, including mitochondrial dysfunction, transcriptional dysregulation, altered protein homeostasis and synaptic dysfunction, which ultimately leads to neuronal cell death [2,5,6,7]. Striatal medium spiny neurons are heavily affected in HD, which contributes to the motor-related symptoms associated with this disorder [8]. In addition to progressive neurodegeneration, patients with HD also experience a range of cognitive, motor, and psychiatric symptoms. Current treatment strategies are limited to the symptomatic management of the disease [9,10].

The prevalence of HD ranges from approximately 5 to 10 cases per 100,000 persons worldwide [11,12]. Over 30,000 people in the United States have HD, with more than 200,000 individuals at risk of inheriting the disease [13,14].

There is an unmet clinical need for disease-modifying therapies for HD, and one of the most promising therapeutic strategies involves targeting HTT at an RNA, DNA, or protein level to lower mHTT, thereby inhibiting the cascade of downstream pathogenic effects [10]. RNA-targeting approaches using antisense oligonucleotides (ASOs) and RNA interference (RNAi) appear to be effective in lowering HTT; however, these therapeutics require direct injection into the central nervous system (CNS) as they cannot penetrate the blood–brain barrier (BBB) [15,16,17,18,19,20]. It is also difficult for these agents to reach deep brain regions such as the caudate and putamen, where neurodegeneration robustly occurs in HD. Additionally, the effects of ASOs are transient, so patients would require perpetual dosing [7,15].

RNAi systems can be delivered using adeno-associated virus (AAV) vectors capable of transfecting cells to enable long-term expression of RNA molecules [21]. The diffusion of AAV’s in the CNS is limited; therefore, direct injections into the brain are often used to overcome this hurdle [22,23].

Direct intrastriatal injection of AAV5-miHTT led to a reduction of *HTT* mRNA in preclinical mice and minipig models of HD [24]. Following on from these findings, UniQure is currently conducting clinical trials in HD patients using this gene therapy known as AMT-130 (NCT05243017 and NCT04120493) [19,24,25]. Results from their 12-month update demonstrate a 53.8% reduction in mHTT in the cerebrospinal fluid (CSF) of early HD patients treated with AMT-130 via intrastriatal delivery [25]. Despite these promising results, this method of delivery is invasive and requires surgery. 

AAV capsids with the ability to cross the BBB, such as AAV9 [26,27], AAV-rh10 [28,29,30], AAV-PHP.B [31,32], and AAV-PHP.eB [33,34], have been discovered or engineered, which enables intravenous administration. However, the transduction efficiency of these capsids can differ between species [35,36] and systemically delivered AAV vectors need to be administered at higher doses in order to achieve therapeutic doses in the brain. Higher doses can cause adverse side effects, including hepatotoxicity and dorsal root ganglion (DRG) toxicity [37,38]. 

Focused ultrasound (FUS) is one approach to overcoming the challenges associated with AAV delivery to the CNS. Application of FUS in combination with microbubbles (MB) can be used to temporarily disrupt the BBB and enable drug delivery to specific brain regions in a less invasive manner [37,38,39]. When systemically circulating microbubbles are exposed to low-pressure ultrasound, they oscillate in the ultrasound field, which exerts mechanical forces on blood vessel walls, leading to increased permeability of the BBB that lasts for several hours. 

The mechanisms through which FUS + MB disrupt the BBB are still being uncovered, but current evidence suggests that BBB opening is achieved through a combination of mechanical and physiological changes [40]. Mechanisms of FUS + MB-induced BBB opening include decreased tight junction protein expression, leading to enhanced paracellular transport, an increased number of intracellular vesicles and endothelial cell openings, enhanced endocytosis, upregulation of transcytosis, and a decrease in the expression of P-glycoprotein [40,41,42,43,44,45]. 

The successful delivery of AAV vectors, antibodies, stem cells, nanotherapeutics, and chemotherapeutics to the brain following the application of FUS + MB has been demonstrated in vivo [46,47,48,49,50]. The expression of reporter genes following the application of FUS + MB has been shown using AAV1, AAV2, AAV1&2, AAV6, AAV8, AAV9, AAVrg, and PHP.B in wild-type mice [46,51,52,53,54] and mouse models of AD [55,56]. The brain regions that have been targeted for AAV delivery using FUS + MB include the cortex, striatum, hippocampus, and thalamus [46,51,52,53,54,56]. Wang et al. (2017) reported the use of FUS-mediated delivery of AAV9-encoding channelrhodopsin-2 (ChR2) in mice, which led to the expression of ChR2 in FUS-targeted brain regions [57]. They also observed more diffuse transgene expression using FUS compared to the direct infusion technique [57].

More recently, Blesa et al. described the use of FUS + MB to deliver modified AAV9 human synapsin vectors (AAV9-PHPeB and AAV9.2-PHPeB) to brain regions associated with PD in non-human primates [58]. Gene delivery with FUS resulted in the expression of green fluorescent protein (GFP) in the putamen and various mid-brain regions. The BBB opening was reversible, safe, and well-tolerated in the NHP’s [58]. 

Reversible FUS-mediated BBB opening has been demonstrated in human patients with glioblastoma tumors, Alzheimer’s disease (AD), Parkinson’s disease (PD), and amyotrophic lateral sclerosis (ALS) in different brain regions, including the prefrontal cortex, entorhinal cortex, hippocampus, and parieto-occipito-temporal cortex, in the absence of adverse side effects [59,60,61,62]. However, no trials have been conducted to assess the safety of FUS-BBB opening in human HD patients. Early clinical trials evaluating the safety of BBB opening in AD, PD, and ALS patients were performed in the absence of any therapeutic drug [59,60,61,62] and there are now ongoing trials delivering antibody therapeutics to AD patients (NCT05469009). The safety of FUS-mediated delivery of gene therapies has not yet been assessed in humans. 

Previous evidence demonstrates that FUS treatment can induce a neuroinflammatory response [47,56,63,64,65,66], and neuroinflammatory responses following direct intracranial and systemic AAV delivery are well documented [67,68,69]. Neuroinflammation is also one hallmark of Huntington’s disease [70,71,72,73,74]. Therefore, before progressing FUS-AAV delivery to the clinic, in the context of HD, it is important to assess whether existing HD pathology could impede delivery or further exacerbate brain pathology following treatment. 

Here, we investigate the FUS-BBB opening for AAV9 delivery in the zQ175 mouse model of HD for the first time. zQ175 mice express the full human *HTT* gene with 175 CAG repeats and replicate some of the neuro-pathophysiology seen in patients, including mHTT aggregation, BBB breakdown, and increased inflammation at later stages of disease progression. The goal of this study was to compare FUS-mediated delivery of ss-AAV9-U6-miR10150-CBA-GFP in healthy wild type mice vs. zQ175 HD model mice at three stages of disease progression: 2 months, 6 months, and 12 months.

## 2. Materials and Methods

### 2.1. Mice and Experimental Design

The animals used in this study are described in Table 1. Heterozygous male and female zQ175 HD mice (B6J.zQ175 KI, JAX Stock #027410) and wild-type controls (C57BL/6J, JAX Stock #000664) were obtained from The Jackson Laboratory (Bar Harbor, ME, USA) at approximately 6–10 weeks of age. All experiments were carried out in accordance with the guidelines and procedures approved by the Brigham and Women’s Hospital Institutional Animal Care and Use Committee (#2016N000103). 

**Figure 1:**
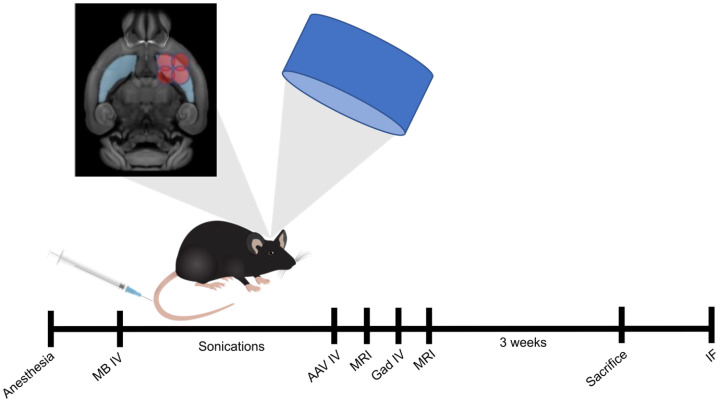
Schematic diagram of FUS-AAV delivery procedure. Mice were treated unilaterally with FUS targeting four regions in the right striatum. Following FUS treatment, mice were injected intravenously with 5.5 × 10^11^ vg/mouse (2.2 × 10^10^ vg/g) of ss-AAV9-U6-miR10150-CBA-GFP and MRI images were acquired before and after gadolinium injections to confirm BBB opening in the targeted brain region. Three weeks after treatment, mice were euthanized, and the brains were cut into coronal sections. The sections were stained for Hoechst (nuclei), GFP, NeuN (neurons), Iba1 (microglia), GFAP (reactive astrocytes), and S100β (astrocytes) to assess GFP area coverage and cell-type specificity of GFP expression. MB, microbubbles; IV, intravenous; MRI, magnetic resonance imaging; Gad, gadolinium; IF, immunofluorescence.

Mice were injected with 5.5 × 10^11^ vg/mouse (2.2 × 10^10^ vg/g) of ss-AAV9-U6-miR10150-CBA-GFP following FUS sonications targeting four focal spots within the right striatum. Then MRI images were acquired to confirm the location and extent of BBB opening (Figure 1). Three weeks following treatment and AAV delivery, the mice were euthanized, and the brains were harvested for immunofluorescence (IF) staining. Analysis was performed to assess delivery efficacy based on the GFP signal and the immune response based on GFAP and Iba1 markers for astrocytosis and microgliosis, respectively. Statistical analysis was performed on all outcome measures to test for significant differences between FUS-targeted and non-targeted brain hemispheres and between mouse groups.

### 2.2. FUS Blood–Brain Barrier Opening and AAV9 Delivery

Mice were anesthetized with a combination of ketamine (Dechra Veterinary Products, Fort Worth, TX, USA, 17033-101-10) and xylazine (Pivetal, Loveland, CO, USA, 21295074) (K = 80/X = 10 mg/kg). The scalp fur was removed with an electric clipper, and depilatory cream was applied to ensure that all the fur was removed to enable sufficient ultrasound coupling. A catheter was inserted into the tail vein for the administration of microbubbles, AAV, and the MRI contrast agent gadolinium (Magnevist, Bayer Healthcare, Whippany, NJ, USA, 88825853). The animal’s head was fixed in a custom-made holder with ear bars for head immobilization and target referencing, which was slotted into an in-house FUS system with the mice in supine position. The FUS system consists of a single-element-focused ultrasound transducer (4 cm diameter, 3.5 cm radius of curvature) operating at 690 kHz, a three-axis manual positioning system, and a passive cavitation detector. The head of the animal was coupled to the transducer with degassed and deionized water.

The sonications were applied using a function generator (33220A, Agilent, Santa Clara, CA, USA), amplifier (240 L, Electronics & Innovation, Rochester, NY, USA), and a customized MATLAB program, which in turn drove the 690 kHz FUS transducer. FUS-mediated BBB opening was targeted to the right striatum, and the parameters used were 10 ms bursts applied at 1 Hz repetition frequency for 120 s at 0.34 MPa [75]. Four sonications were applied sequentially to the striatum based on previously established coordinates over a 2 × 2 mm square to ensure maximum coverage of the target region [76]. The FUS sonications were performed immediately after the intravenous administration of microbubbles (100 μL/kg bolus injection of Optison (GE Healthcare, Chicago, IL, USA, 2707-03). The AAV was injected immediately after sonications via the tail-vein catheter at 5.5 × 10^11^ vector genomes (vg)/mouse (approximately 2.2 × 10^10^ vg/g based on 25 g mouse body weight).

### 2.3. Magnetic Resonance Imaging

T1-weighted contrast enhanced MRI images were acquired using 7T (WT, 2-, and 6-month zQ175 mice) and 3T (12-month zQ175 mice) preclinical MRI scanners (Bruker, Karlsruhe, Germany) to confirm the region of BBB opening and assess the level of BBB disruption. Following BBB opening, the mouse was removed from the FUS system and placed in a Bruker animal holder with the nose of the animal inserted into a nose cone for the delivery of isoflurane gas and oxygen. The breathing rate of the animal was monitored throughout the MRI procedure. T1-weighted contrast-enhanced MRI images were acquired pre- and post-bolus injection of gadolinium (Magnevist, Bayer Healthcare, Whippany, NJ, USA, 88825853), 0.25 mL/kg) and converted to percent difference. After the MRI procedure, the mice were allowed to recover on heat pads and housed for the prescribed time period until euthanization. Due to images being acquired at a different main field strength, MRI results for the 12-month zQ175 mice are presented but were excluded from statistical analysis.

### 2.4. AAV Construct

**Figure 2:**
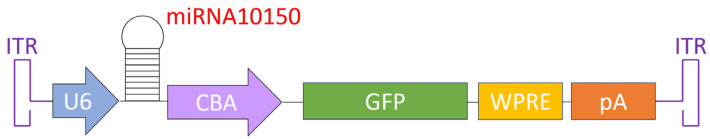
Diagram of AAV9-U6-miRNA10150-CBA-GFP construct.

The ss-AAV9-U6-miR10150-CBA-GFP virus was kindly donated to us by Dr. Miguel Sena-Esteves and Dr. Ana Rita Batista for use in this study. The vector contains a U6 promoter, which drives the transcription of miR10150 targeting bp10150 in the 3′ untranslated region of the mouse and human HTT gene, and the chimeric cytomegalovirus enhancer/chicken β-actin (CBA) promoter, which drives the expression of GFP (Figure 2). While this study was not designed to assess the efficacy of HTT lowering, the construct containing the micro-RNA, miR10150, was used in this study in order to assess the safety of the full construct.

### 2.5. Brain Perfusion and Tissue Processing

Animals were euthanized three weeks after FUS sonications, and then the mouse brains were harvested for histological analysis. The mice were anesthetized with isoflurane and transcardially perfused with 1× PBS, followed by 4% paraformaldehyde (PFA) solution in PBS. The brains were then transferred to slotted cassettes and incubated overnight in 4% PBS/PFA solution at 4 °C. After three washes with cold PBS, the tissues were stored in 1× PBS at 4 °C until they were required for staining. 

For sectioning, the brains were mounted on the vibratome stage using cyanoacrylate glue and sectioned into 30 µm coronal sections using the Leica VT 1000 S (Leica, Wetzlar, Germany) vibratome. The free-floating sections were stored in PBS with 0.05% sodium azide (Teknova, Hollister, CA, USA, S0209) at 4 °C until processing.

### 2.6. Immunofluorescence Staining

Coronal floating sections were incubated in blocking solution (1% [*w*/*v*] bovine serum albumin (Sigma-Aldrich, St. Louis, MO, USA, A2153-50G), 5% normal goat serum [*v*/*v*] (Jackson ImmunoResearch Laboratories, West Grove, PA, USA, 005-00-121), and 0.2% Triton X-100 [*v*/*v*]) (Sigma-Aldrich, St. Louis, MO, USA, X100-500ML) for 1 h at room temperature. Sections were washed three times with 0.05% phosphate-buffered saline-Tween (PBS-T) and incubated in 300 µL of primary antibody diluted in blocking buffer for 2 nights at 4 °C. Tissues were then washed four times in 0.05% PBS-T and incubated with 300 µL of secondary antibody and Hoechst 33342 (Fisher Scientific, Hampton, NH, USA, 62249) (1:1000) diluted in blocking buffer for 2 h at room temperature. Sections were washed four times in 0.05% PBS-T before mounting onto Superfrost Plus microscope slides (Fisher Scientific, 1255015) with.

Mounted sections were air-dried and cover-slipped with Prolong Gold Antifade Reagent (Invitrogen, Waltham, MA, USA, P36930). Primary antibody dilutions: chicken anti-GFP 1:200 (Invitrogen, A10262), rabbit anti-NeuN 1:200 (MilliporeSigma, Burlington, MA, USA, ABN78), mouse anti-S100β 1:200 (MilliporeSigma, Burlington, MA, USA, S2657), rabbit anti-GFAP 1:2500 (MilliporeSigma, AB5804), rabbit anti-Iba1 1:1000 (Wako Chemicals USA, Richmond, VA, USA, 016-20001). Secondary antibody dilutions: Goat anti-rabbit IgG (H + L) (Alexa fluor 647) (Invitrogen, Waltham, MA, USA, A32733), goat anti-chicken IgY (H + L) (Alexa fluor 555) (Invitrogen, Waltham, MA, USA, A32932), goat anti-mouse Cy5 (Jackson ImmunoResearch Laboratories, West Grove, PA, USA, 115-175-146). All secondary antibodies were diluted 1:500. 

### 2.7. Quantification of BBB Opening Area

**Figure 3:**
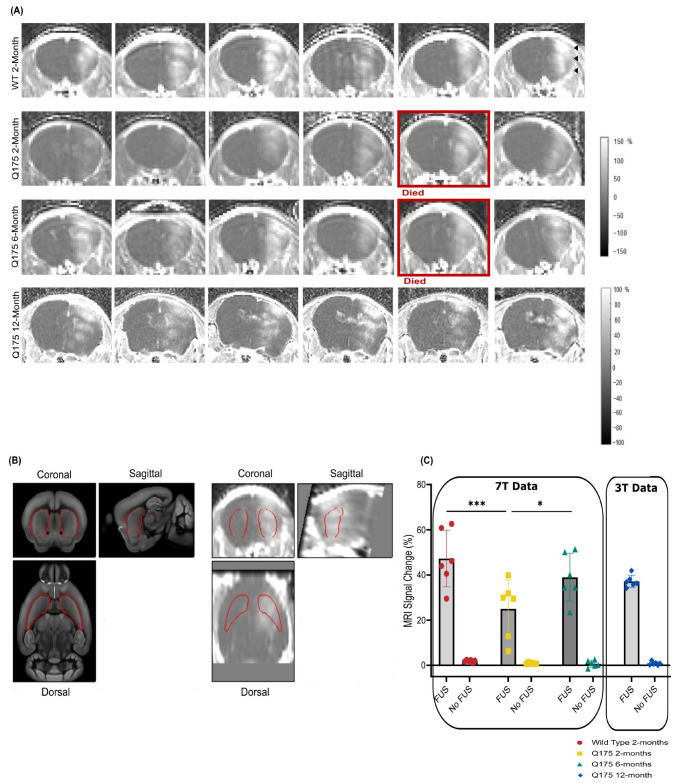
BBB opening confirmation with T1-weighted contrast-enhanced MRI. (**A**) Gadolinium-enhanced T1-weighted coronal images (presented as percent change before and after gadolinium injections) showing the location and extent of BBB opening for each animal in the WT 2-month, zQ175 2-month, and zQ175 6-month-old groups, acquired with a 7T MRI scanner. The images presented for the zQ175 12-month cohort were acquired with a 3T scanner. The MRI images demonstrate an increase in BBB permeability in the FUS-treated hemisphere (indicated with black arrows) compared to the untreated hemisphere. (**B**) The Gad-enhanced T1-weighted images were co-registered to a mouse brain template to determine percent signal change in the striatum (outlined in red). (**C**) Percent signal change in the striatum of WT 2-month (*n* = 6), zQ175 2-month (*n* = 6), zQ175 6-month (*n* = 6), and zQ175 12-month-old mice (*n* = 6). Two-way ANOVA and Tukey’s post hoc test for multiple comparisons were used for statistical analysis. * *p* < 0.05, *** *p* < 0.001.

For BBB opening quantification, the T1-weighted contrast MRI images were manually co-registered to an MRI mouse brain template using affine transformations [76]. Pre- and post-contrast injection images were converted to percent signal change. The signal percent change was averaged over pre-defined regions of interest (ROIs) from the mouse brain atlas for the left and right striatum (see Figure 3B) [76].

### 2.8. Quantification of GFP Area and GFAP Fluorescence Intensity

For GFP area quantification, images of whole brain sections were acquired using the Olympus VS120 slide scanner microscope (Olympus America Inc., Center Valley, PA, USA) with a 20× objective. Images were acquired using three fluorescent filters: DAPI (Hoechst), TRITC (GFP), and cy5 (NeuN/GFAP/s100β). The images were then analyzed with a custom MATLAB script for quantification of the GFP area. The MATLAB image processing library was used to draw ROIs for the right (FUS-targeted) and left (non-targeted) hemispheres. A threshold was set for the GFP signal, and the area containing the GFP signal above the threshold was calculated in each hemisphere. GFP area coverage was determined in six WT, five zQ175 2-month, five zQ175 6-month mice, and six zQ175 12-month mice. 

Twelve sections per mouse were imaged to quantify the area covered by GFP in each hemisphere. The GFP area for each hemisphere was then averaged across the twelve sections and converted into percent of hemisphere covered. 

GFAP fluorescence intensity was quantified as a measure of the level of astrocyte activation. The GFAP images acquired at 20×, as described above, were used. ROIs were drawn over the entire left and right striatum and the left and right cortex. Pixel values within the ROIs were summed and divided by the number of pixels in the ROI to generate an integrated density value. Integrated density values were calculated for all ROIs in three sections per mouse and averaged over the sections.

### 2.9. Quantification of Cell Type Transduction

The percentage of neurons or astrocytes expressing GFP was quantified in six WT, five zQ175 2-month, five zQ175 6-month mice, and six zQ175 12-month mice using three sections per animal. For quantification of GFP-positive neurons or astrocytes, two ROIs in the cortex and striatum were imaged across 3 sections per mouse stained with GFP, Hoechst, and NeuN or S100β using the Zeiss LSM 880 confocal microscope (Carl Zeiss GmbH, Jena, Germany) and a 20× objective. The ROIs were selected based on areas with high GFP expression in the FUS-targeted hemisphere. Neuron and astrocyte counts were done manually by one blinded observer using the Image J software version 1.54f (NIH, Bethesda, MD, USA) cell counter plugin. Hoechst stains the nuclei of all cells and provides a total cell count. Percentages of transduced neurons and astrocytes were calculated by dividing the number of NeuN^+^ or S100β^+^ cells that co-localized with Hoechst and GFP by the total number of GFP^+^ cells multiplied by 100. The percentages of GFP^+^ cells that were neurons or astrocytes were calculated as the percentage of GFP^+^/NeuN^+^/Hoechst^+^ cells or GFP^+^/S100β^+^/Hoechst^+^ cells out of the total number of Hoechst^+^ cells and multiplied by 100. These percentages were calculated for two ROIs each in the cortex and striatum for each section per mouse and averaged across the two ROIs and the three sections.

### 2.10. Statistical Analysis

GraphPad Prism 10 (GraphPad Software Inc., CA, USA) was used for statistical analysis. Statistical tests were carried out in GraphPad Prism using a two-way ANOVA framework. F-tests were performed to test for significant effects of the main factors (e.g., mouse group and FUS/no FUS). When a significant main effect was found, post hoc tests with correction for multiple comparisons were performed to test for significant differences between individual groups. Significant results are reported as: * *p* < 0.05, ** *p* < 0.01, *** *p* < 0.001, **** *p* < 0.0001.

## 3. Results

### 3.1. T1-Weighted Contrast-Enhanced MRI Images Reveal BBB Disruption in Targeted Brain Regions

BBB opening was confirmed with T1-weighted contrast-enhanced MR images following FUS treatment and AAV administration. Gadolinium entry was observed in the FUS-treated hemisphere in all mice (Figure 3A), and two-way ANOVA analysis of MRI signal change demonstrated a significant difference in BBB permeability between the FUS-treated hemisphere and the contralateral side in WT 2-month, zQ175 2-month, and zQ175 6-month mice (*p* < 0.0001). There was also a significant difference in BBB permeability between the three experimental groups imaged at 7T (*p* = 0.0082). Post-hoc tests indicated significantly higher BBB permeability in the treated hemisphere of WT 2-month-old mice compared to zQ175 2-month-old mice (*p* < 0.0003). BBB permeability was also significantly higher in zQ175 6-month-old mice compared to the zQ175 2-month-old group (*p* = 0.0211; Figure 3C). BBB opening was also confirmed in the zQ175 12-month cohort imaged at 3T; however, the data set from this group was excluded from statistical analysis due to the fact that these images were acquired with a 3T scanner. Note that 1 mouse from the zQ175 2-month group and 1 mouse from the zQ175 6-month group died on the day of treatment following MR imaging due to unknown causes. Overall, the remaining mice recovered normally from surgery and thrived until their euthanasia at the end of the experiment.

### 3.2. FUS-Mediated AAV9 Delivery Restricted Transgene Expression to Sonicated Hemisphere

**Figure 4:**
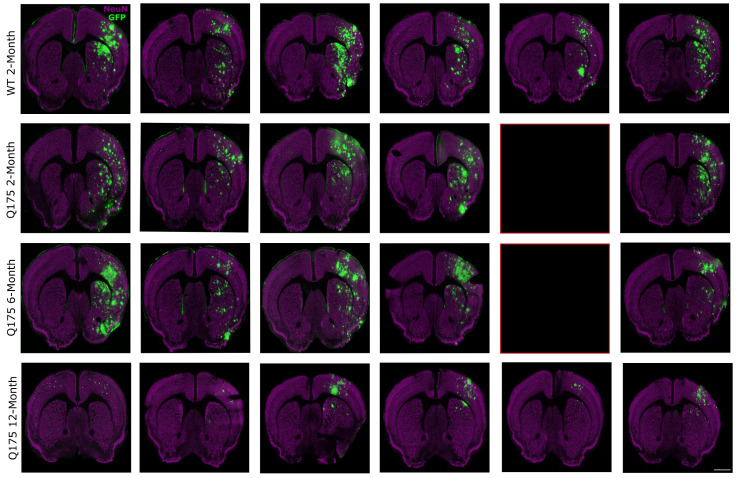
FUS treatment enhances gene delivery in sonicated hemisphere. Mice were treated with FUS targeting four areas in the right striatum and then injected with ss-AAV9-U6-miR10150-CBA-GFP intravenously. Coronal sections stained for NeuN (purple), GFP (green), and Hoechst (not shown) represent one section for each FUS-treated mouse. GFP expression was detected in the cortex and striatum of the FUS-treated hemisphere by immunofluorescence. The red boxes indicate missing sections due to the death of mice zQ175_11 and zQ175_5. Magnification, 20×; scale bar represents 500 µm. WT 2-month (*n* = 6), zQ175 2-month (*n* = 5), zQ175 6-month (*n* = 5), and zQ175 12-month (*n* = 6).

**Figure 5:**
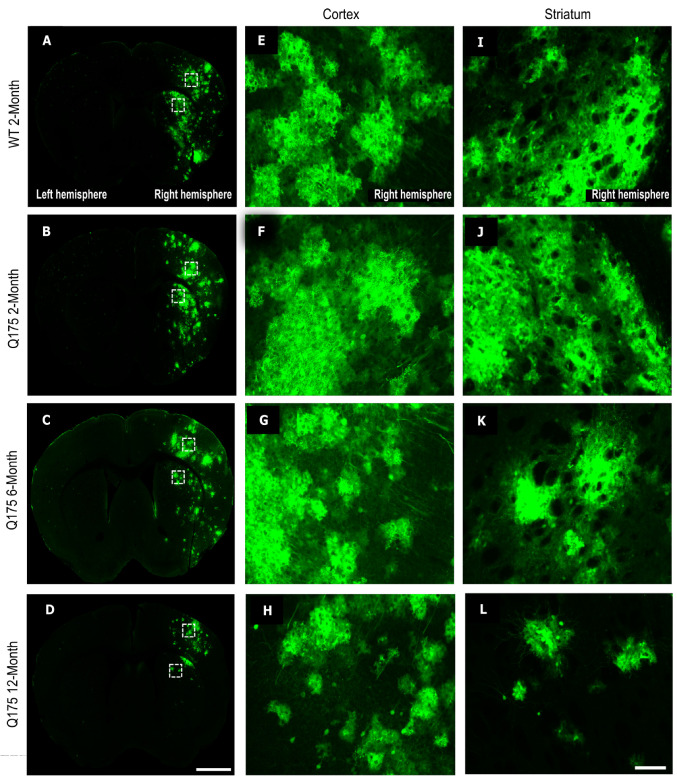
GFP expression in the striatum and cortex following FUS treatment. Representative coronal sections of heterogenous GFP expression in WT 2-month, zQ175 2-month, zQ175 6-month, and zQ175 12-month-old mouse brains following FUS treatment and intravenous injection of ss-AAV9-U6-miR10150-CBA-GFP. The dashed white boxes indicate regions of interest (ROIs) in the cortex and striatum that were zoomed into for figures (**E**–**L**). (**A**–**D**) Example whole-brain images showing GFP expression in the cortex and striatum inside the sonicated hemisphere of WT and zQ175 HD mice; Magnification, 20×. (**E**–**H**) Zoomed ROI of GFP expression in the FUS-treated cortex of WT 2-month, zQ175 2-month, zQ175 6-month, and zQ175 12-month mice; (**I**–**L**) zoomed ROI of GFP expression in the FUS-treated striatum of WT 2-month, zQ175 2-month, zQ175 6-month, and zQ175 12-month-old mice. Scale bar represents 500 µm (**A**–**D**) and 100 µm (**E**–**L**).

GFP expression was almost entirely restricted to the FUS-treated hemisphere. Strong GFP expression was visible in the targeted striatum and in the cortex of the treated hemisphere of WT 2-month, zQ175 2-month, and zQ175 6-month-old mice (Figure 4 and Figure 5), generally following the pattern of signal increase seen on the contrast MRI images. GFP expression was weaker in the treated hemisphere of zQ175 12-month mice compared to the other cohorts. There was also limited GFP expression within the FUS-treated striatum in this group (Figure 4). Virtually no GFP expression was detected in most of the animals within the contralateral hemisphere, which served as an internal control.

**Figure 6:**
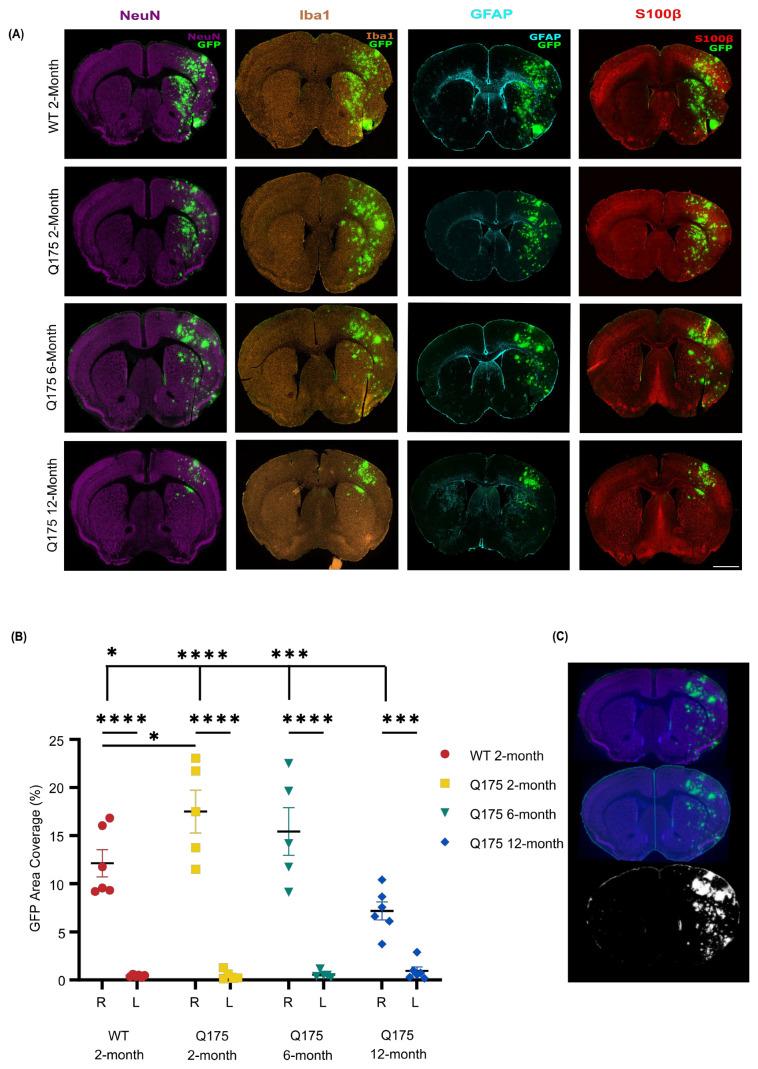
FUS-mediated delivery of ss-AAV9-U6-miR10150-CBA-GFP. (**A**) Representative images of whole-brain coronal sections stained for GFP (green), NeuN (purple), Iba1 (brown), GFAP (cyan), and S100β (red) from WT 2-month, zQ175 2-month, zQ175 6-month, and zQ175 12-month-old mice following FUS treatment and intravenous administration of ss-AAV9-U6-miR10150-CBA-GFP at 5.5 *×* 10^11^ vg/mouse. Magnification, 20×; scale bar represents 500 µm. (**B**) Quantification of GFP coverage by the percent area of each hemisphere with GFP signal above threshold. WT 2-month (*n* = 6), zQ175 2-month (*n* = 5), zQ175 6-month (*n* = 5) and zQ175 12-month (*n* = 6). Data were analyzed using a two-way ANOVA followed by Tukey’s multiple comparisons test. * *p* < 0.05, *** *p* < 0.001, ****p < 0.0001. (**C**) Analysis of GFP area coverage using an image processing script in MATLAB. An ROI was drawn around each hemisphere, then the two hemispheres were overlayed with a mask, and then % GFP area was quantified in each hemisphere based on GFP signal above threshold.

The area of the GFP signal above threshold was quantified in each hemisphere from sections stained for GFP (green), NeuN (purple), Iba1 (brown), GFAP (cyan), and S100β (red) (Figure 6A). Results demonstrate a significantly higher expression of GFP in the FUS-treated side compared to the contralateral side in the WT 2-month (*p* < 0.0001), zQ175 2-month (*p* < 0.0001), zQ175 6-month (*p* < 0.0001), and zQ175 12-month-old groups (*p* = 0.0008) (Figure 6B). GFP expression was also significantly higher in the cortex of zQ175 2-month mice compared to the WT cohort (*p* = 0.0236). The expression of GFP was significantly lower in the zQ175 12-month group compared to WT 2-month (*p* = 0.0313), zQ175 2-month (*p* =< 0.0001), and zQ175 6-month (*p* = 0.0003)-old mice.

### 3.3. FUS-Mediated Delivery of AAV9-U6-miR10150-CBA-GFP Leads to Primarily Neuronal and Secondarily Astrocytic Transduction

**Figure 7:**
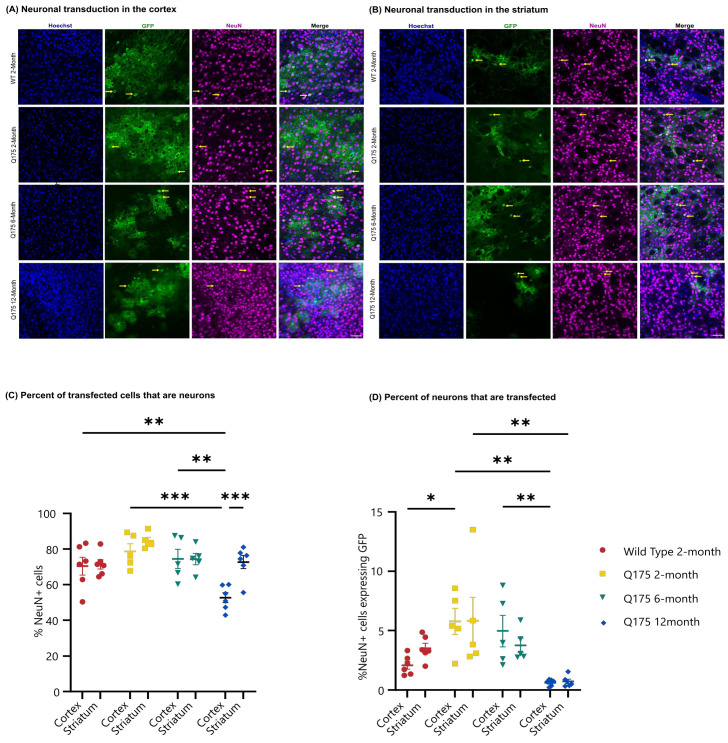
GFP expression in neurons in the FUS-treated hemisphere following AAV administration. Confocal microscopic analysis of Hoechst (blue), GFP (green), and NeuN (magenta) expression in (**A**) the cortex and (**B**) striatum of the FUS-treated hemisphere of WT 2-month, zQ175 2-month, zQ175 6-month and zQ175 12-month-old mice injected intravenously with ss-AAV9-U6-miR10150-CBA-GFP at 5.5 *×* 10^11^ vg/mouse. Yellow arrows highlight examples of colocalization of GFP (green) with the neuronal marker NeuN (magenta). Scale bar represents 50 µm. (**C**) Percentage of GFP^+^ cells that are neurons in the striatum and cortex. (**D**) Percentage of Hoechst cells that are GFP^+^ and NeuN^+^ in the striatum and cortex. WT 2-month (*n* = 6), zQ175 2-month (*n* = 5), zQ175 6-month (*n* = 5), and zQ175 12-month (*n* = 6). Data were analyzed using a two-way ANOVA followed by Tukey’s multiple comparisons test. * *p* < 0.05, ** *p* < 0.01, *** *p* < 0.001. Data shown are mean ± SEM.

**Figure 8:**
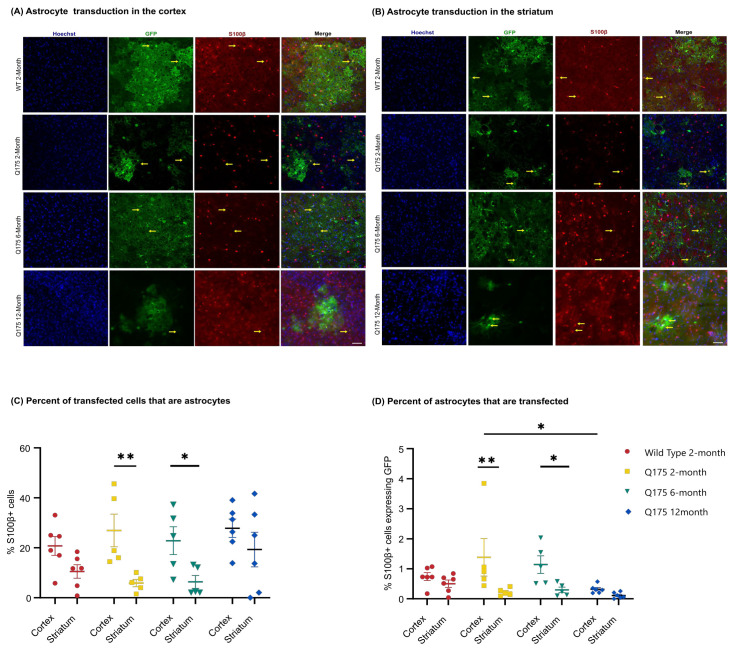
GFP expression in astrocytes in the FUS-treated hemisphere following AAV administration. Confocal microscopic analysis of Hoechst (blue), GFP (green), and S100β (red) staining in the (**A**) cortex and (**B**) striatum of the FUS-treated hemisphere of WT 2-month, zQ175 2-month, zQ175 6-month, and zQ175 12-month-old mice injected intravenously with ss-AAV9-U6-miR10150-CBA-GFP at 5.5 *×* 10^11^ vg/mouse. Yellow arrows highlight colocalization of GFP (green) with the astrocytic marker S100β (red). Scale bar represents 50 µm. (**C**) Percentage of GFP^+^ cells that are astrocytes in the striatum and cortex. (**D**) Percentage of Hoechst^+^ cells that are GFP^+^ and S100b^+^ cells in the striatum and cortex. WT 2-month (*n* = 6), zQ175 2-month (*n* = 5), zQ175 6-month (*n* = 5), and zQ175 12-month (*n* = 6)-old mice. Data were analyzed using a two-way ANOVA followed by Tukey’s multiple comparisons test. * *p* < 0.05, ** *p* < 0.01. Data shown are mean ± SEM.

Cell type transduction was determined in the striatum and cortex of the FUS-treated side using confocal images to determine the colocalization of GFP expression with anti-NeuN (Figure 7A,B) as a marker of neurons or anti-S100β (Figure 8A,B) as an astrocytic marker. FUS-mediated delivery of AAV9-U6-miR10150 resulted in 0.2–13% of neurons and 0.04–4% of astrocytes being transduced. Of the cell types measured, ~70% of GFP^+^ cells were neurons (Figure 7C), ~25% of GFP-positive cells were astrocytes (Figure 8C), and ~5% of cells were NeuN^−^ and S100β^−^. We did not observe any signs of neuronal loss, and there were no significant differences in the number of NeuN^+^ cells in the cortex or striatum of WT or HD mice in regions with high GFP expression.

The proportion of GFP^+^ cells that were labeled as neurons was fairly consistent across all mouse groups and the two brain regions considered, with the exception of the cortex in the zQ175 12-month mice. While other mouse groups had an average proportion of GFP^+^ cells in the range of 70% to 85%, the zQ175 12-month showed 73% in the striatum but only 53% in the cortex. This was significantly lower than the percentage in the striatum for the same mice (*p* = 0.0004) and significantly lower than the percentages in the cortex for the WT 2-month (*p* = 0.0071), zQ175 2-month (*p* = 0.0001), and zQ175 6-month (*p* = 0.0013) (Figure 7C). 

The two main significant differences seen in the percentage of neurons transduced are in line with the results of GFP coverage. The zQ175 2-month mice had significantly higher neuronal transduction in the cortex at 6% compared to 2% in the cortex of WT 2-month mice (*p* = 0.0288), and the zQ175 12-month mice had the lowest level of neuronal transduction in both cortex and striatum at 0.6% and 0.7%, respectively. These values are significantly lower compared to the zQ175 2-month (cortex and striatum) and 6-month groups (cortex only). There were no significant differences in neuronal transduction levels between the striatum and cortex across all four experimental groups. 

There was a significant difference in the proportion of GFP^+^ cells that were labeled as astrocytes between the cortex and striatum in zQ175 2-month (*p* = 0.0036) and zQ175 6-month (*p* = 0.0199) mice (Figure 8C). The transduction of astrocytes was significantly higher in the cortex compared to the striatum in zQ175 2-month (*p* = 0.0019) and zQ175 6-month (*p* = 0.0202)-old mice (Figure 8D). There was also a higher percentage of astrocytes transduced in the cortex compared to the striatum in WT 2-month mice: Cortex (0.74 ± 0.13), striatum (0.50 ± 0.12), and zQ175 12-month mice: Cortex (0.32 ± 0.06), striatum (0.11 ± 0.04), but these differences were not significant (Figure 8D). Significantly fewer astrocytes were transduced in the cortex of zQ175 12-month mice compared to the zQ175 2-month group (*p* = 0.0147) (Figure 8D). 

### 3.4. FUS-Mediated Delivery of AAV9-U6-miR10150-CBA-GFP Induces Mild Astrocytosis

**Figure 9:**
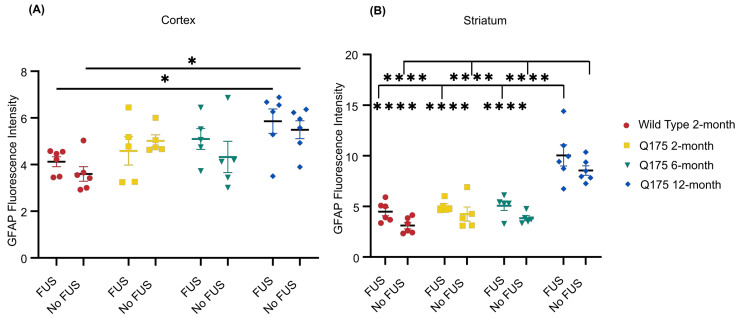
GFAP fluorescence intensity. (**A**) Quantification of GFAP fluorescence in the cortex and (**B**) striatum of FUS-treated WT 2-month (*n* = 6), zQ175 2-month (*n* = 5), zQ175 6-month (*n* = 5), and zQ175 12-month (*n* = 6) mice following AAV delivery. * *p* < 0.05, **** *p* < 0.0001. Data shown are mean ± SEM.

Quantification of GFAP fluorescence intensity revealed slightly higher average GFAP signal intensity in the FUS-treated hemisphere for all four mouse groups in the striatum and for three of the four groups in the cortex (Figure 9). Two-way ANOVA analysis indicated a significant main effect on GFAP intensity was present for FUS vs. no FUS in the striatum. However, significant differences were not seen within any individual groups after applying Tukey’s multiple comparisons test (Figure 9). 

GFAP intensity was significantly elevated in the zQ175 12-month mice. In the cortex, both sonicated (*p* = 0.0308) and non-sonicated (*p* = 0.0159) hemispheres had significantly stronger GFAP signal intensity than WT 2-month mice. In the striatum, GFAP signal intensity in both hemispheres was significantly stronger compared to all other mouse groups (*p* < 0.0001). 

### 3.5. Minimal Microglial Activation following FUS-Mediated Delivery of AAV9-U6-miR10150-CBA-GFP

**Figure 10:**
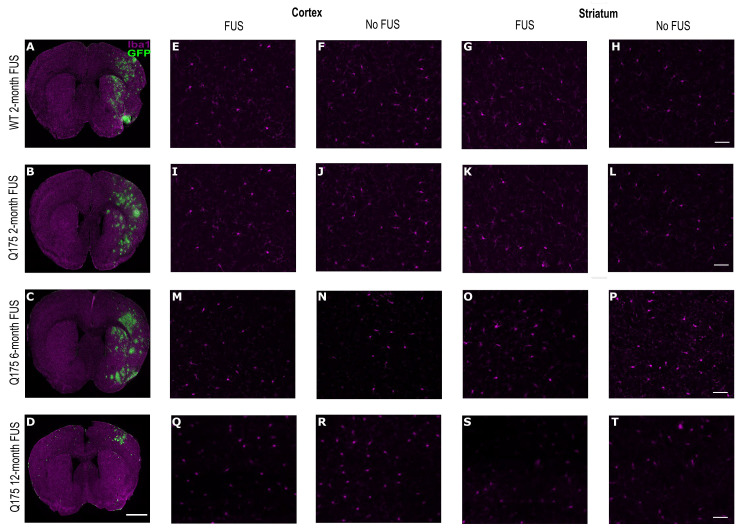
Iba1 expression in the cortex and striatum of WT and HD mice after 3 weeks following treatment. (**A**–**D**) Representative whole-brain images of Iba1 (magenta) and GFP staining for each experimental group. Magnification 20. Confocal microscopic analysis of Iba1 (magenta) staining in the cortex and striatum of the FUS-treated hemisphere and contralateral side in (**A**,**E**–**H**) WT 2-month, (**B**,**I**–**L**) zQ175 2-month, (**C**,**M**–**P**) zQ175 6-month, and (**D**,**Q**–**T**) zQ175 12-month mice. Scale bar represents 500 µm for (**A**–**D**) and 50 µm for the rest.

Overall, there were minimal differences in microglial activation in the cortex and striatum between the FUS-treated hemisphere and contralateral side. In a few instances, there were small foci at the dorsal striatum just under the corpus collosum that showed morphologies consistent with reactive microglia in areas with high GFP expression. There were no obvious differences in Iba1 signal intensity or the morphology and density of Iba1^+^ cells between the four different experimental groups (Figure 10). 

## 4. Discussion

### 4.1. FUS-Mediated Delivery of AAV’s Increases AAV Transduction Area

Gene therapy using adeno-associated viral vectors has emerged as a promising strategy to treat neurodegenerative diseases, including Huntington’s disease. However, delivery is a formidable challenge when it comes to administering gene therapies due to the BBB [77,78,79,80]. 

An advantage of FUS over direct injections is an increase in AAV transduction area [54]. We observed a significantly greater area of GFP coverage in the sonicated hemisphere compared to the contralateral side in all four experimental groups. GFP expression was almost exclusively restricted to the FUS-treated hemisphere, even though AAV9 has the ability to cross the BBB following intravenous injection [19,25]. This may be due to the fact that the dose used in this study was not sufficient to induce neuronal transduction through intravenous injection alone. Thus, focused ultrasound enhanced the systemic delivery of ss-AAV9-U6-miR10150-CBA-GFP. These findings are in line with previous studies demonstrating that FUS improves the delivery of AAV vectors [46,51,52,54,56].

### 4.2. HD Disease State May Influence FUS-Mediated AAV Delivery

Pathology in the CNS due to Huntington’s disease can manifest as increased levels of neuroinflammatory markers and possibly increased BBB permeability. In the zQ175 mouse model of HD, mHTT aggregates, some motor deficits, and a reduction in cortical and striatal brain volumes are all seen by 6 months [81,82]. The main goal of this study was to determine whether this pathology would affect the FUS-mediated delivery of AAVs to the brain.

Interestingly, we did see a significant reduction in the level of BBB disruption induced by FUS + MB treatment in the younger cohort of HD mice, as measured by gadolinium-based contrast MRI. However, there were no significant differences in BBB disruption between WT mice and the 6-month HD mice in which disease pathology would be more advanced. This difference in the level of BBB disruption did not translate to differences in GFP expression. Despite a significantly lower level of BBB disruption in the zQ175 2-month group, there was a significantly higher area of GFP coverage in this younger HD cohort compared to the WT group. There were also significant differences between mouse groups in terms of the percent of transfected cells that were neurons, with a significantly higher percentage of GFP^+^ neurons in the cortex of zQ175 2-month mice compared to WT. The neuronal transduction efficiencies within the cortex of zQ175 2-month and zQ175 6-month mice were also significantly higher compared to the oldest zQ175 12-month cohort. There was a significantly higher percentage of transduced neurons in the FUS-treated striatum of zQ175 2-month vs. zQ175 12-month mice. Neuronal transduction efficiencies were also higher in this younger HD group compared to the 6-month and WT groups, but the differences were not significant. 

Quantification of astrocyte activation revealed significantly higher levels of GFAP expression in the FUS-treated and un-treated striatum of zQ175 12-month mice compared to the zQ175 6-month, zQ175 2-month, and WT groups. The higher levels of astrocytic activation in the oldest HD cohort are in line with previous studies that demonstrate that zQ175 mice exhibit increased astrocyte pathology at later stages of disease progression [83]. All mouse groups had, on average, higher GFAP expression in the FUS-targeted striatum compared to the non-targeted striatum, but this difference did not rise to the level of significance within individual groups. Together, these safety and delivery efficacy measurements indicate that HD pathology, as it exists in 12-month-old zQ175 mice, may influence FUS-mediated delivery of AAV vectors to the brain.

### 4.3. FUS-Mediated AAV Delivery Has the Potential to Lower Systemic AAV Doses

Systemic administration of AAV vectors often requires higher doses of vector to achieve therapeutic concentrations in the brain [77,84]. Higher vector doses are associated with an increased incidence of hepatotoxicity [85,86].

FUS-mediated delivery of AAVs that can cross the BBB to targeted brain regions can be achieved using doses that are 10–100 lower than conventional doses required to transduce brain cells [46,63,87,88]. In this present study, neuronal transduction was achieved with a relatively low dose of AAV, 2.2 × 10^10^ vg/g = 2.2 × 10^13^ vg/kg, compared to what has been tested in the clinic (ranging from ~10^11^–10^14^ vg/kg per patient) [35,89,90]. The dose used in this study is approximately ten times lower than the dose used in the X-linked myotubular myopathy (XLMTM) trial, in which three patients in the high-dose arm died after developing severe hepatotoxicity [91,92,93]. This supports the existing evidence showing that focused ultrasound can be used to deliver lower doses of AAV to targeted brain regions [46,53,63]. Developing new strategies to enable the delivery of lower AAV doses is essential in reducing the incidence of adverse events and AAV-toxicity-related deaths in gene therapy clinical trials. 

### 4.4. Non-Invasive Gene Delivery Using FUS Results in Neuronal and Astrocytic Transduction

The FUS-mediated delivery of ss-AAV9-U6-miR10150-CBA-GFP led to the transduction of neurons and astrocytes in wild-type and zQ175 HD mice. We observed greater neuronal tropism, with 53% or more of the cells transduced representing neurons, compared to 6–28% representing astrocytes. The intracerebroventricular delivery of AAV9 in mice has also been shown to result in the preferential transduction of neurons [19,94]. Intravascular delivery of ssAAV9 or scAAV9 without the use of FUS BBB opening can result in variable transduction efficiencies in neurons and astrocytes in mice [25,87]. Tropism following systemic AAV9 delivery can also differ between brain regions [19,87]. Gray et al. reported twice as many neurons being transduced in the striatum as astrocytes following intravenous injection of scAAV9/CBh-GFP [87]. They also reported that the transduction efficiency following intravascular delivery of ssAAV9/CMV-GFP at 5 × 10^11^ vg (2.5 × 10^13^ vg/kg) was lower compared to scAAV9 vectors, and the transduction efficiency was similar to that observed with a 20-fold lower (2.5 × 10^10^ vg) dose of scAAV9 [87]. 

The transduction of neurons and astrocytes following FUS-mediated delivery of AAV9 to the CNS has been demonstrated by several groups [46,54,56,63]. In a study comparing the transduction efficiency of AAV1, AAV2, AAV5, AAV9, and AAVrg following FUS treatment, AAV9 transduced the highest percentage of neurons and astrocytes in the striatum [54]. However, the proportion of GFP^+^ cells that are neurons or astrocytes in FUS-targeted brain regions varies between studies, depending on the region targeted. Thevenot et al. observed a greater proportion of astrocytes (63%) were transduced compared to neurons (18%) in the striatum of C57BL/6 mice following FUS-mediated delivery of scAAV9-CB-GFP (2.5 × 10^9^ vg/g) [46]. Whereas the percentage of neurons (58%) transduced in the hippocampus was higher compared to astrocytes (36%) [46]. Kofoed et al. have demonstrated transduction efficiencies of up to ~7% in neurons and ~20% in astrocytes within FUS-targeted brain regions using AAV9 with transgene expression under the control of CAG and CBA promoters [54,56]. Direct comparisons of transduction efficiencies are difficult between studies due to differences in animal model, AAV serotype, promoter, brain region, FUS parameters, AAV dose, age at time of injection, and cell type quantification methods [19,52,53,54,56]. However, our data support the existing literature demonstrating that AAVs can be delivered using focused ultrasound to selected brain regions. 

There is no consensus on how many neurons need to be transduced in order to produce therapeutic benefits, and the percentage of transgene-positive neurons is not always reported in preclinical gene therapy studies. The IV delivery of an AAV9 based gene replacement therapy using the Mecp2 knockout model of Rett syndrome improved survival in the treated cohort compared to the untreated Mecp2-null mice, despite relatively low levels of neuronal transduction (2–4%) [95]. In another study, parkinsonian NHPs that received intrastriatal infusions of AAV2-hAADC demonstrated long-term clinical improvements, with only 5–6% of neurons expressing the AADC transgene [96]. These findings suggest that transgene expression in 2–6% of neurons may be sufficient to produce therapeutic effects.

### 4.5. Neuroinflammatory Responses following FUS Treatment and Systemic AAV Administration

AAV delivery to the CNS can induce a neuroinflammatory response, especially when administering higher doses. This response is marked by an up-regulation of GFAP and Iba1 expression [67,68,69]. 

Neuroinflammatory responses following FUS-BBB opening have also been reported in FUS-targeted brain regions with and without AAV delivery [56,63,64,65,66,97]. A vast majority of FUS-AAV studies have been performed in WT mice, but here we treated zQ175 mice at different ages. These mice show upregulated GFAP expression as HD progresses in this model [83]. Increased GFAP and Iba1 expression have been noted following FUS + MB-mediated BBB opening [64,65,66,97]. In some cases, this immune response resolved within days, but other studies report a persistent increase in GFAP and Iba1 immunoreactivity up to 7 weeks after treatment [65,97]. The degree of inflammation observed following FUS-BBB opening is related to different factors, including microbubble dose and FUS pressure [98,99,100,101].

Here, we observed that the FUS-mediated delivery of ss-AAV9-U6-miR10150-CBA-GFP led to a non-significant increase in GFAP intensity within the FUS-treated striatum compared to the contralateral side three weeks following treatment. Kofoed et al. also reported increased GFAP expression within the FUS-targeted striatum, three weeks after FUS-mediated delivery of AAV9 and AAV2-HBKO [63]. 

GFAP immunoreactivity needs to be evaluated at later time points (i.e., 3-, 6-, and 9-months) following FUS-AAV delivery to determine potential long-term inflammation. It has also been demonstrated that AAV9 vectors encoding non-self proteins such as GFP can trigger immune responses that are associated with the activation of astrocytes and microglia [69]. Therefore, for the long-term assessment of FUS-AAV neuroinflammation, it will be important to conduct studies without reporter genes that could elicit an immune response.

### 4.6. Limitations of the Study

Although we demonstrate that FUS can be used to safely deliver an AAV9-based gene therapy in the zQ175 model of HD, this study has some limitations. Firstly, we used a geometrically focused single-element transducer, which resulted in an elliptical focal spot spanning the cortex and striatum. The focal size could be reduced by using a dual-crossed ultrasound transducer system to improve targeting accuracy. We used a coordinate-based approach to target the striatum, which does not account for anatomical variability between animals or variations in animal position with respect to the ultrasound transducer. Adding MR- or ultrasound-based guidance would improve target repeatability. 

Another limitation of the study design is that we did not include WT 6-month and 12-month mice to enable age-matched comparisons, as we were primarily interested in assessing the impact of HD progression on FUS-mediated AAV delivery.

We chose AAV9 because it is widely considered the gold standard for targeting the CNS after systemic delivery and has been extensively used to transduce cells of the central nervous system in mice. Neuronal transduction was achieved in our study, but at low levels. Newer engineered capsids have been shown to have higher transduction efficiencies compared to AAV9. AAV transduction following FUS-mediated delivery could be improved further by using capsids with enhanced CNS transduction or capsids that have been optimized for acoustic delivery.

Even though the miRNA was included in the construct to assess safety, we did not evaluate therapeutic efficacy. Now that the delivery and safety efficacy have been evaluated, it will be important to assess the therapeutic benefits of this treatment in future studies. 

## 5. Conclusions

Here we demonstrated for the first time in a mouse model of HD that FUS could be used to disrupt the BBB and deliver the viral vector AAV9-U6-miR10150-CBA-GFP using intravenous injection at a relatively low dose to the striatum and cortex. Gene delivery resulted in strong GFP expression within the striatum and cortex in the sonicated hemisphere, at a systemic dose where virtually no expression was detected on the contralateral side. Neurons and astrocytes were infected, with a higher percentage of neurons being transduced in the FUS-treated striatum compared to astrocytes. There were minimal differences in the safety and delivery efficacy profiles between the WT mice and the younger two groups of HD mice. However, delivery efficacy was markedly reduced in the older 12-month HD mice. Interestingly, it was also at this age that we observed an increase in baseline astrocytosis, but this underlying inflammation was not exacerbated by the FUS and gene therapy treatment. 

This preclinical study shows that delivery and safety following FUS treatment are similar in 2-month, and 6-month-old HD mice compared to WT. Overall, the results generated from this study provide a basis for future studies to investigate the therapeutic benefits of FUS-mediated delivery of this ss-AAV9 construct containing the micro-RNA, miR10150 targeting HTT transcripts for degradation.

## Figures and Tables

**Table 1:** Summary of experimental groups.

Group	*n*	Mouse Strain	Age at Treatment	FUS Target
1. WT 2-month	6	C57BL/6J	2 months	Right caudate putamen
2. zQ175 2-month	6 *	B6J.zQ175 KI	2 months	Right caudate putamen
3. zQ175 6-month	6 ^†^	B6J.zQ175 KI	6 months	Right caudate putamen
4. zQ175 12-month	6	B6J.zQ175 KI	12 months	Right caudate putamen

* One mouse in group 2 died after MRI imaging, leaving *n* = 5 for histological analysis. ^†^ One mouse in group 3 died after MRI imaging, leaving *n* = 5 for histological analysis.

## Data Availability

Data are available upon request.

## Abbreviations

AAV, adeno-associated virus; ASO, anti-sense oligonucleotide; BBB, blood–brain barrier; CAG, cytosine–adenine–guanine; cDNA, complementary deoxyribonucleic acid; ChR2, channelrhodopsin-2; CNS, central nervous system; DNA, deoxyribonucleic acid; DRG, dorsal root ganglion; FUS, focused ultrasound; Gad, gadolinium; GFP, green fluorescent protein; HD, Huntington’s disease; IF, immunofluorescence; IV, intravenous; MB, microbubbles; mHTT, mutant huntingtin; miRNA, micro ribonucleic acid; MRI, magnetic resonance imaging; mRNA, messenger ribonucleic acid; RNA, ribonucleic acid; RNAi, ribonucleic acid interference; ROI, region of interest; WT, wild-type.

## References

[1] The Huntington’s Disease Collaborative Research Group (1993). A novel gene containing a trinucleotide repeat that is expanded and unstable on Huntington’s disease chromosomes. Cell.

[2] Rubinsztein D.C., Leggo J., Coles R., Almqvist E., Biancalana V., Cassiman J.J., Chotai K., Connarty M., Crauford D., Curtis A. (1996). Phenotypic characterization of individuals with 30–40 CAG repeats in the Huntington disease (HD) gene reveals HD cases with 36 repeats and apparently normal elderly individuals with 36–39 repeats. Am. J. Hum Genet..

[3] Bates G.P., Dorsey R., Gusella J.F., Hayden M.R., Kay C., Leavitt B.R., Nance M., Ross C.A., Scahill R.I., Wetzel R. (2015). Huntington disease. Nat. Rev. Dis. Primers.

[4] Rojas N.G., Cesarini M.E., Peker G., Da Prat G.A., Etcheverry J.L., Gatto E.M. (2022). Review of Huntington’s Disease: From Basics to Advances in Diagnosis and Treatment. J. Neurol. Res..

[5] DiFiglia M., Sapp E., Chase K.O., Davies S.W., Bates G.P., Vonsattel J.P., Aronin N. (1997). Aggregation of huntingtin in neuronal intranuclear inclusions and dystrophic neurites in brain. Science.

[6] Gutekunst C.-A., Li S.-H., Yi H., Mulroy J.S., Kuemmerle S., Jones R., Rye D., Ferrante R.J., Hersch S.M., Li X.-J. (1999). Nuclear and neuropil aggregates in Huntington’s disease: Relationship to neuropathology. J. Neurosci..

[7] Tabrizi S.J., Ghosh R., Leavitt B.R. (2019). Huntingtin lowering strategies for disease modification in Huntington’s disease. Neuron.

[8] Bergonzoni G., Döring J., Biagioli M. (2021). D1R-and D2R-medium-sized spiny neurons diversity: Insights into striatal vulnerability to huntington’s disease mutation. Front. Cell. Neurosci..

[9] Keum J.W., Shin A., Gillis T., Mysore J.S., Abu Elneel K., Lucente D., Hadzi T., Holmans P., Jones L., Orth M. (2016). The HTT CAG-expansion mutation determines age at death but not disease duration in Huntington disease. Am. J. Hum. Genet..

[10] Ferguson M.W., Kennedy C.J., Palpagama T.H., Waldvogel H.J., Faull R.L.M., Kwakowsky A. (2022). Current and possible future therapeutic options for Huntington’s disease. J. Cent. Nerv. Syst. Dis..

[11] Medina A., Mahjoub Y., Shaver L., Pringsheim T. (2022). Prevalence and incidence of Huntington’s disease: An updated systematic review and meta-analysis. Mov. Disord..

[12] Shafie A., Ashour A.A., Anjum F., Shamsi A., Hassan M.I. (2024). Elucidating the Impact of Deleterious Mutations on IGHG1 and Their Association with Huntington’s Disease. J. Pers. Med..

[13] Komatsu H. (2021). Innovative therapeutic approaches for Huntington’s disease: From nucleic acids to GPCR-targeting small molecules. Front. Cell. Neurosci..

[14] Kordasiewicz H.B., Stanek L.M., Wancewicz E.V., Mazur C., McAlonis M.M., Pytel K.A., Artates J.W., Weiss A., Cheng S.H., Shihabuddin L.S. (2012). Sustained therapeutic reversal of Huntington’s disease by transient repression of huntingtin synthesis. Neuron.

[15] Southwell A.L., Kordasiewicz H.B., Langbehn D., Skotte N.H., Parsons M.P., Villanueva E.B., Caron N.S., Ostergaard M.E., Anderson L.M., Xie Y. (2018). Huntingtin suppression restores cognitive function in a mouse model of Huntington’s disease. Sci. Transl. Med..

[16] Tabrizi S.J., Leavitt B.R., Landwehrmeyer G.B., Wild E.J., Saft C., Barker R.A., Blair N.F., Craufurd D., Priller J., Rickards H. (2019). Targeting huntingtin expression in patients with Huntington’s disease. N. Engl. J. Med..

[17] Boudreau R.L., McBride J.L., Martins I., Shen S., Xing Y., Carter B.J., Davidson B.L. (2009). Nonallele-specific silencing of mutant and wild-type huntingtin demonstrates therapeutic efficacy in Huntington’s disease mice. Mol. Ther..

[18] McBride J.L., Pitzer M.R., Boudreau R.L., Dufour B., Hobbs T., Ojeda S.R., Davidson B.L. (2011). Preclinical safety of RNAi-mediated HTT suppression in the rhesus macaque as a potential therapy for Huntington’s disease. Mol. Ther..

[19] Foust K.D., Nurre E., Montgomery C.L., Hernandez A., Chan C.M., Kaspar B.K. (2009). Intravascular AAV9 preferentially targets neonatal neurons and adult astrocytes. Nat. Biotechnol..

[20] Meijer D.H., Maguire C.A., LeRoy S.G., Sena-Esteves M. (2009). Controlling brain tumor growth by intraventricular administration of an AAV vector encoding IFN-β. Cancer Gene Ther..

[21] Xu X., Chen W., Zhu W., Chen J., Ma B., Ding J., Wang Z., Li Y., Wang Y., Zhang X. (2021). Adeno-associated virus (AAV)-based gene therapy for glioblastoma. Cancer Cell Int..

[22] Evers M.M., Miniarikova J., Juhas S., Valles A., Bohuslavova B., Juhasova J., Skalnikova H.K., Vodicka P., Valekova I., Brouwers C. (2018). AAV5-miHTT gene therapy demonstrates broad distribution and strong human mutant huntingtin lowering in a Huntington’s disease minipig model. Mol. Ther..

[23] Spronck E.A., Brouwers C.C., Vallès A., de Haan M., Petry H., van Deventer S.J., Konstantinova P., Evers M.M. (2019). AAV5-miHTT gene therapy demonstrates sustained huntingtin lowering and functional improvement in Huntington disease mouse models. Mol. Ther. Methods Clin. Dev..

[24] Estevez-Fraga C., Tabrizi S.J., Wild E.J. (2022). Huntington’s Disease Clinical Trials Corner: November 2022. J. Huntington’s Dis..

[25] Duque S., Joussemet B., Riviere C., Marais T., Dubreil L., Douar A.-M., Fyfe J., Moullier P., Colle M.-A., Barkats M. (2009). Intravenous administration of self-complementary AAV9 enables transgene delivery to adult motor neurons. Mol. Ther..

[26] Gao G.-P., Alvira M.R., Wang L., Calcedo R., Johnston J., Wilson J.M. (2002). Novel adeno-associated viruses from rhesus monkeys as vectors for human gene therapy. Proc. Natl. Acad. Sci. USA.

[27] Gao G., Alvira M.R., Somanathan S., Lu Y., Vandenberghe L.H., Rux J.J., Calcedo R., Sanmiguel J., Abbas Z., Wilson J.M. (2003). Adeno-associated viruses undergo substantial evolution in primates during natural infections. Proc. Natl. Acad. Sci. USA.

[28] Tanguy Y., Biferi M.G., Besse A., Astord S., Cohen-Tannoudji M., Marais T., Barkats M. (2015). Systemic AAVrh10 provides higher transgene expression than AAV9 in the brain and the spinal cord of neonatal mice. Front. Mol. Neurosci..

[29] Deverman B.E., Pravdo P.L., Simpson B.P., Kumar S.R., Chan K.Y., Banerjee A., Wu W.-L., Yang B., Huber N., Pasca S.P. (2016). Cre-dependent selection yields AAV variants for widespread gene transfer to the adult brain. Nat. Biotechnol..

[30] Hordeaux J., Yuan Y., Clark P.M., Wang Q., Martino R.A., Sims J.J., Bell P., Raymond A., Stanford W.L., Wilson J.M. (2019). The GPI-linked protein LY6A drives AAV-PHP.B transport across the blood-brain barrier. Mol. Ther..

[31] Chan K.Y., Jang M.J., Yoo B.B., Greenbaum A., Ravi N., Wu W.-L., Sánchez-Guardado L., Lois C., Mazmanian S.K., Deverman B.E. (2017). Engineered AAVs for efficient noninvasive gene delivery to the central and peripheral nervous systems. Nat. Neurosci..

[32] Mathiesen S.N., Lock J.L., Schoderboeck L., Abraham W.C., Hughes S.M. (2020). CNS transduction benefits of AAV-PHP.eB over AAV9 are dependent on administration route and mouse strain. Mol. Ther. Methods Clin. Dev..

[33] Hordeaux J., Wang Q., Katz N., Buza E.L., Bell P., Wilson J.M. (2018). The neurotropic properties of AAV-PHP.B are limited to C57BL/6J mice. Mol. Ther..

[34] Matsuzaki Y., Konno A., Mochizuki R., Shinohara Y., Nitta K., Okada Y., Hirai H. (2018). Intravenous administration of the adeno-associated virus-PHP.B capsid fails to upregulate transduction efficiency in the marmoset brain. Neurosci. Lett..

[35] Mendell J.R., Al-Zaidy S., Shell R., Arnold W.D., Rodino-Klapac L.R., Prior T.W., Lowes L., Alfano L., Berry K., Church K. (2017). Single-dose gene-replacement therapy for spinal muscular atrophy. N. Engl. J. Med..

[36] Buss N., Lanigan L., Zeller J., Cissell D., Metea M., Adams E., Higgins M., Kim K.H., Budzynski E., Yang L. (2022). Characterization of AAV-mediated dorsal root ganglionopathy. Mol. Ther. Methods Clin. Dev..

[37] Hynynen K., McDannold N., Vykhodtseva N., Jolesz F.A. (2001). Noninvasive MR imaging–guided focal opening of the blood-brain barrier in rabbits. Radiology.

[38] Raymond S.B., Treat L.H., Dewey J.D., McDannold N.J., Hynynen K., Bacskai B.J. (2008). Ultrasound enhanced delivery of molecular imaging and therapeutic agents in Alzheimer’s disease mouse models. PLoS ONE.

[39] Konofagou E.E., Tunga Y.-S., Choia J., Deffieuxa T., Baseria B., Vlachosa F. (2012). Ultrasound-induced blood-brain barrier opening. Curr. Pharm. Biotechnol..

[40] Wasielewska J.M., White A.R. (2022). Focused Ultrasound-mediated Drug Delivery in Humans—A Path Towards Translation in Neurodegenerative Diseases. Pharm. Res..

[41] Sheikov N., McDannold N., Sharma S., Hynynen K. (2008). Effect of focused ultrasound applied with an ultrasound contrast agent on the tight junctional integrity of the brain microvascular endothelium. Ultrasound Med. Biol..

[42] Cho H., Lee H.-Y., Han M., Choi J.-R., Ahn S., Lee T., Chang Y., Park J. (2016). Localized down-regulation of P-glycoprotein by focused ultrasound and microbubbles induced blood-brain barrier disruption in rat brain. Sci. Rep..

[43] Olsman M., Sereti V., Mühlenpfordt M., Johnsen K.B., Andresen T.L., Urquhart A.J., Davies C.d.L. (2021). Focused ultrasound and microbubble treatment increases delivery of transferrin receptor-targeting liposomes to the brain. Ultrasound Med. Biol..

[44] Mungur R., Zheng J., Wang B., Chen X., Zhan R., Tong Y. (2022). Low-intensity focused ultrasound technique in glioblastoma multiforme treatment. Front. Oncol..

[45] Sheikov N., McDannold N., Vykhodtseva N., Jolesz F., Hynynen K. (2004). Cellular mechanisms of the blood-brain barrier opening induced by ultrasound in presence of microbubbles. Ultrasound Med. Biol..

[46] Thévenot E., Jordão J.F., O’Reilly M.A., Markham K., Weng Y.-Q., Foust K.D., Kaspar B.K., Hynynen K., Aubert I. (2012). Targeted delivery of self-complementary adeno-associated virus serotype 9 to the brain, using magnetic resonance imaging-guided focused ultrasound. Hum. Gene Ther..

[47] Jordão J.F., Ayala-Grosso C.A., Markham K., Huang Y., Chopra R., McLaurin J., Hynynen K., Aubert I. (2010). Antibodies targeted to the brain with image-guided focused ultrasound reduces amyloid-β plaque load in the TgCRND8 mouse model of Alzheimer’s disease. PLoS ONE.

[48] Burgess A., Ayala-Grosso C.A., Ganguly M., Jordão J.F., Aubert I., Hynynen K. (2011). Targeted delivery of neural stem cells to the brain using MRI-guided focused ultrasound to disrupt the blood-brain barrier. PLoS ONE.

[49] Ohta S., Kikuchi E., Ishijima A., Azuma T., Sakuma I., Ito T. (2020). Investigating the optimum size of nanoparticles for their delivery into the brain assisted by focused ultrasound-induced blood–brain barrier opening. Sci. Rep..

[50] Liu H.-L., Hua M.-Y., Chen P.-Y., Chu P.-C., Pan C.-H., Yang H.-W., Huang C.-Y., Wang J.-J., Yen T.-C., Wei K.-C. (2010). Blood-brain barrier disruption with focused ultrasound enhances delivery of chemotherapeutic drugs for glioblastoma treatment. Radiology.

[51] Hsu P.-H., Wei K.-C., Huang C.-Y., Wen C.-J., Yen T.-C., Liu C.-L., Lin Y.-T., Chen J.-C., Shen C.-R. (2013). Noninvasive and targeted gene delivery into the brain using microbubble-facilitated focused ultrasound. PLoS ONE.

[52] Wang S., Olumolade O.O., Sun T., Samiotaki G., Konofagou E.E. (2015). Noninvasive, neuron-specific gene therapy can be facilitated by focused ultrasound and recombinant adeno-associated virus. Gene Ther..

[53] Weber-Adrian D., Kofoed R.H., Silburt J., Noroozian Z., Shah K., Burgess A., Rideout S., Kügler S., Hynynen K., Aubert I. (2021). Systemic AAV6-synapsin-GFP administration results in lower liver biodistribution, compared to AAV1&2 and AAV9, with neuronal expression following ultrasound-mediated brain delivery. Sci. Rep..

[54] Kofoed R.H., Dibia C.L., Noseworthy K., Xhima K., Vacaresse N., Hynynen K., Aubert I. (2022). Efficacy of gene delivery to the brain using AAV and ultrasound depends on serotypes and brain areas. J. Control. Release.

[55] Weber-Adrian D., Kofoed R.H., Chan J.W.Y., Silburt J., Noroozian Z., Kügler S., Hynynen K., Aubert I. (2019). Strategy to enhance transgene expression in proximity of amyloid plaques in a mouse model of Alzheimer’s disease. Theranostics.

[56] Kofoed R.H., Heinen S., Silburt J., Dubey S., Dibia C.L., Maes M., Simpson E.M., Hynynen K., Aubert I. (2021). Transgene distribution and immune response after ultrasound delivery of rAAV9 and PHP.B to the brain in a mouse model of amyloidosis. Mol. Ther. Methods Clin. Dev..

[57] Wang S., Kugelman T., Buch A., Herman M., Han Y., Karakatsani M.E., Hussaini S.A., Duff K., Konofagou E.E. (2017). Non-invasive, focused ultrasound-facilitated gene delivery for optogenetics. Sci. Rep..

[58] Blesa J., Pineda-Pardo J.A., Inoue K.-I., Gasca-Salas C., Balzano T., Del Rey N.L.-G., Reinares-Sebastián A., Esteban-García N., Rodríguez-Rojas R., Márquez R. (2023). BBBB opening with focused ultrasound in nonhuman primates and Parkinson’s disease patients: Targeted AAV vector delivery and PET imaging. Sci. Adv..

[59] Rezai A.R., Ranjan M., D’haese P.-F., Haut M.W., Carpenter J., Najib U., Mehta R.I., Chazen J.L., Zibly Z., Yates J.R. (2020). Noninvasive hippocampal blood−brain barrier opening in Alzheimer’s disease with focused ultrasound. Proc. Natl. Acad. Sci. USA.

[60] Lipsman N., Meng Y., Bethune A.J., Huang Y., Lam B., Masellis M., Herrmann N., Heyn C., Aubert I., Boutet A. (2018). Blood–brain barrier opening in Alzheimer’s disease using MR-guided focused ultrasound. Nat. Commun..

[61] Gasca-Salas C., Fernandez-Rodriguez B., Pineda-Pardo J.A., Rodriguez-Rojas R., Obeso I., Hernandez-Fernandez F., del Alamo M., Mata D., Guida P., Ordas-Bandera C. (2021). Blood-brain barrier opening with focused ultrasound in Parkinson’s disease dementia. Nat. Commun..

[62] Abrahao A., Meng Y., Llinas M., Huang Y., Hamani C., Mainprize T., Aubert I., Heyn C., Black S.E., Hynynen K. (2019). First-in-human trial of blood–brain barrier opening in amyotrophic lateral sclerosis using MR-guided focused ultrasound. Nat. Commun..

[63] Kofoed R.H., Noseworthy K., Wu K., Sivadas S., Stanek L., Elmer B., Hynynen K., Shihabuddin L.S., Aubert I. (2022). The engineered AAV2-HBKO promotes non-invasive gene delivery to large brain regions beyond ultrasound targeted sites. Mol. Ther. Methods Clin. Dev..

[64] Kovacs Z.I., Burks S.R., Frank J.A. (2018). Focused ultrasound with microbubbles induces sterile inflammatory response proportional to the blood brain barrier opening: Attention to experimental conditions. Theranostics.

[65] Kovacs Z.I., Tu T.-W., Sundby M., Qureshi F., Lewis B.K., Jikaria N., Burks S.R., Frank J.A. (2018). MRI and histological evaluation of pulsed focused ultrasound and microbubbles treatment effects in the brain. Theranostics.

[66] Sinharay S., Tu T.-W., Kovacs Z.I., Schreiber-Stainthorp W., Sundby M., Zhang X., Papadakis G.Z., Reid W.C., Frank J.A., Hammoud D.A. (2019). In vivo imaging of sterile microglial activation in rat brain after disrupting the blood-brain barrier with pulsed focused ultrasound:[18F] DPA-714 PET study. J. Neuroinflamm..

[67] Reimsnider S., Manfredsson F.P., Muzyczka N., Mandel R.J. (2007). Time course of transgene expression after intrastriatal pseudotyped rAAV2/1, rAAV2/2, rAAV2/5, and rAAV2/8 transduction in the rat. Mol. Ther..

[68] Lowenstein P.R., Mandel R.J., Xiong W., Kroeger K., Castro M.G. (2007). Immune responses to adenovirus and adeno-associated vectors used for gene therapy of brain diseases: The role of immunological synapses in understanding the cell biology of neuroimmune interactions. Curr. Gene Ther..

[69] Samaranch L., Sebastian W.S., Kells A.P., Salegio E.A., Heller G., Bringas J.R., Pivirotto P., DeArmond S., Forsayeth J., Bankiewicz K.S. (2014). AAV9-mediated expression of a non-self protein in nonhuman primate central nervous system triggers widespread neuroinflammation driven by antigen-presenting cell transduction. Mol. Ther..

[70] Tai Y.F., Pavese N., Gerhard A., Tabrizi S.J., Barker R.A., Brooks D.J., Piccini P. (2007). Microglial activation in presymptomatic Huntington’s disease gene carriers. Brain.

[71] Tai Y.F., Pavese N., Gerhard A., Tabrizi S.J., Barker R.A., Brooks D.J., Piccini P. (2007). Imaging microglial activation in Huntington’s disease. Brain Res. Bull..

[72] Politis M., Lahiri N., Niccolini F., Su P., Wu K., Giannetti P., Scahill R.I., Turkheimer F.E., Tabrizi S.J., Piccini P. (2015). Increased central microglial activation associated with peripheral cytokine levels in premanifest Huntington’s disease gene carriers. Neurobiol. Dis..

[73] Crotti A., Glass C.K. (2015). The choreography of neuroinflammation in Huntington’s disease. Trends Immunol..

[74] Lois C., González I., Izquierdo-García D., Zürcher N.R., Wilkens P., Loggia M.L., Hooker J.M., Rosas H.D. (2018). Neuroinflammation in Huntington’s disease: New insights with 11C-PBR28 PET/MRI. ACS Chem. Neurosci..

[75] Todd N., Zhang Y., Livingstone M., Borsook D., McDannold N. (2019). The neurovascular response is attenuated by focused ultrasound-mediated disruption of the blood-brain barrier. Neuroimage.

[76] Paxinos G., Franklin K.B.J. (2001). The Mouse Brain in Stereotaxic Coordinates.

[77] Pardridge W.M. (2020). Blood-brain barrier and delivery of protein and gene therapeutics to brain. Front. Aging Neurosci..

[78] Marchi P.M., Marrone L., Azzouz M. (2022). Delivery of therapeutic AAV9 vectors via cisterna magna to treat neurological disorders. Trends Mol. Med..

[79] Tardieu M., Zérah M., Gougeon M.L., Ausseil J., de Bournonville S., Husson B., Zafeiriou D., Parenti G., Bourget P., Poirier B. (2017). Intracerebral gene therapy in children with mucopolysaccharidosis type IIIB syndrome: An uncontrolled phase 1/2 clinical trial. Lancet Neurol..

[80] Fiandaca M.S., Forsayeth J.R., Dickinson P.J., Bankiewicz K.S. (2008). Image-guided convection-enhanced delivery platform in the treatment of neurological diseases. Neurotherapeutics.

[81] Heikkinen T., Lehtimäki K., Vartiainen N., Puoliväli J., Hendricks S.J., Glaser J.R., Bradaia A., Wadel K., Touller C., Kontkanen O. (2012). Characterization of neurophysiological and behavioral changes, MRI brain volumetry and 1H MRS in zQ175 knock-in mouse model of Huntington’s disease. PLoS ONE.

[82] Menalled L.B., Kudwa A.E., Miller S., Fitzpatrick J., Watson-Johnson J., Keating N., Ruiz M., Mushlin R., Alosio W., McConnell K. (2012). Comprehensive behavioral and molecular characterization of a new knock-in mouse model of Huntington’s disease: zQ175. PLoS ONE.

[83] Brown T.G., Thayer M.N., VanTreeck J.G., Zarate N., Hart D.W., Heilbronner S., Gomez-Pastor R. (2023). Striatal spatial heterogeneity, clustering, and white matter association of GFAP+ astrocytes in a mouse model of Huntington’s disease. Front. Cell. Neurosci..

[84] Perez B.A., Shutterly A., Chan Y.K., Byrne B.J., Corti M. (2020). Management of neuroinflammatory responses to AAV-mediated gene therapies for neurodegenerative diseases. Brain Sci..

[85] Agarwal S. (2020). High-dose AAV gene therapy deaths. Nat. Biotechnol..

[86] Chand D., Mohr F., McMillan H., Tukov F.F., Montgomery K., Kleyn A., Sun R., Tauscher-Wisniewski S., Kaufmann P., Kullak-Ublick G. (2021). Hepatotoxicity following administration of onasemnogene abeparvovec (AVXS-101) for the treatment of spinal muscular atrophy. J. Hepatol..

[87] Gray S.J., Matagne V., Bachaboina L., Yadav S., Ojeda S.R., Samulski R.J. (2011). Preclinical differences of intravascular AAV9 delivery to neurons and glia: A comparative study of adult mice and nonhuman primates. Mol. Ther..

[88] Noroozian Z., Xhima K., Huang Y., Kaspar B.K., Kügler S., Hynynen K., Aubert I. (2019). MRI-guided focused ultrasound for targeted delivery of rAAV to the brain. Adeno-Associated Virus Vectors: Design and Delivery.

[89] Au H.K.E., Isalan M., Mielcarek M. (2022). Gene therapy advances: A meta-analysis of AAV usage in clinical settings. Front. Med..

[90] Maurya S., Sarangi P., Jayandharan G.R. (2022). Safety of Adeno-associated virus-based vector-mediated gene therapy—Impact of vector dose. Cancer Gene Ther..

[91] Shieh P.B., Kuntz N., Smith B., Bonnemann C.G., Dowling J.J., Lawlor M.W., Mueller-Felber W., Noursalehi M., Rico S., Servais L. (2019). ASPIRO phase 1/2 gene therapy trial in X-linked myotubular myopathy (XLMTM): Update on preliminary safety and efficacy findings. Mol. Ther..

[92] Shieh P.B., Bönnemann C.G., Müller-Felber W., Blaschek A., Dowling J.J., Kuntz N.L., Seferian A.M. (2020). Re: “Moving Forward after Two Deaths in a Gene Therapy Trial of Myotubular Myopathy” by Wilson and Flotte. Human Gene Ther..

[93] Kishimoto T.K., Samulski R.J. (2022). Addressing high dose AAV toxicity–‘one and done’ or ‘slower and lower’?. Expert Opin. Biol. Ther..

[94] Cearley C.N., Vandenberghe L.H., Parente M.K., Carnish E.R., Wilson J.M., Wolfe J.H. (2008). Expanded repertoire of AAV vector serotypes mediate unique patterns of transduction in mouse brain. Mol. Ther..

[95] Gadalla K.K., Bailey M.E., Spike R.C., Ross P.D., Woodard K.T., Kalburgi S.N., Bachaboina L., Deng J.V., West A.E., Samulski R.J. (2013). Improved survival and reduced phenotypic severity following AAV9/MECP2 gene transfer to neonatal and juvenile male Mecp2 knockout mice. Mol. Ther..

[96] Hadaczek P., Eberling J.L., Pivirotto P., Bringas J., Forsayeth J., Bankiewicz K.S. (2010). Eight years of clinical improvement in MPTP-lesioned primates after gene therapy with AAV2-hAADC. Mol. Ther..

[97] Jordão J.F., Thévenot E., Markham-Coultes K., Scarcelli T., Weng Y.-Q., Xhima K., O’Reilly M., Huang Y., McLaurin J., Hynynen K. (2013). Amyloid-β plaque reduction, endogenous antibody delivery and glial activation by brain-targeted, transcranial focused ultrasound. Exp. Neurol..

[98] Todd N., Angolano C., Ferran C., Devor A., Borsook D., McDannold N. (2020). Secondary effects on brain physiology caused by focused ultrasound-mediated disruption of the blood–brain barrier. J. Control. Release.

[99] Tsai H.-C., Tsai C.-H., Chen W.-S., Inserra C., Wei K.-C., Liu H.-L. (2018). Safety evaluation of frequent application of microbubble-enhanced focused ultrasound blood-brain-barrier opening. Sci. Rep..

[100] Choi H.J., Han M., Seo H., Park C.Y., Lee E.-H., Park J. (2022). The new insight into the inflammatory response following focused ultrasound-mediated blood–brain barrier disruption. Fluids Barriers CNS.

[101] McMahon D., Hynynen K. (2017). Acute inflammatory response following increased blood-brain barrier permeability induced by focused ultrasound is dependent on microbubble dose. Theranostics.

